# *Elaeagnus umbellata* Fruit Extract Protects Skin from Ultraviolet-Mediated Photoaging in Hairless Mice

**DOI:** 10.3390/antiox13020195

**Published:** 2024-02-03

**Authors:** Seok-Man Park, Cheol-Jong Jung, Dae-Geon Lee, Yeong-Eun Yu, Tae-Hun Ku, Mu-Seok Hong, Tae-Kyung Lim, Kwong-Il Paeng, Hyun-Ki Cho, Il-Je Cho, Sae-Kwang Ku

**Affiliations:** 1 Department of Histology and Anatomy, College of Korean Medicine, Daegu Haany University, Gyeongsan 38610, Republic of Korea; smpark@okchundang.co.kr (S.-M.P.); oc_cjjung@okchundang.co.kr (C.-J.J.); ghost71715@okchundang.co.kr (D.-G.L.); 2 Central Research Center, Okchundang Inc., Daegu 41059, Republic of Korea; youye@okchundang.co.kr; 3 Okchundang Korean Medicine Clinic, Ulsan 44900, Republic of Korea; oc100002@okchundang.co.kr; 4 Rodam Korean Medical Clinic, Seoul 06038, Republic of Korea; hongms@skincora.com (M.-S.H.); hltkn@skincora.com (T.-K.L.); rodamca@skincora.com (K.-I.P.); wasavihg@skincora.com (H.-K.C.)

**Keywords:** *Elaeagnus umbellata* fruit extract, Elaea, ultraviolet ray, skin photoaging, antioxidant, anti-inflammation

## Abstract

Photoaging refers to the accumulation of skin damage which includes wrinkle formation, loss of elasticity, and epidermal thickening due to repeated ultraviolet (UV) irradiation. The present study investigated the protective effects of *Elaeagnus umbellata* fruit extract (Elaea) on UV-mediated photoaged skin of SKH1 hairless mice and compared the effects of Elaea with ascorbic acid. Although there was no difference in body weight between groups during experimental period, oral administration of 50–200 mg/kg Elaea once daily for 15 weeks significantly prevented an increase in skin weight, epithelial thickening of epidermis, and apoptosis caused by UV irradiation. Skin replica and histopathological analyses revealed that Elaea dose-dependently decreased wrinkle and microfold formation. In addition, Elaea administration restored UV-mediated reduction in type I collagen and hyaluronan through the inhibition of matrix metalloproteinases and p38 mitogen-activated protein kinase expression. Moreover, Elaea suppressed UV-dependent increases in superoxide anion production, fatty acid oxidation, and protein nitration by up-regulating antioxidant system. Furthermore, Elaea alleviated infiltration of inflammatory cells in UV-irradiated skin. The preventive effects of 100 mg/kg Elaea administration against UV-induced photoaging were similar to those by 100 mg/kg ascorbic acid. Collectively, the present study suggests that the *E. umbellata* fruit is a promising edible candidate to prevent skin photoaging.

## 1. Introduction

Skin is the largest organ which maintains homeostasis by protecting the human body from sunlight, environmental pollutants, microorganisms, and other factors. The outermost stratum corneum, composed of multilayered keratinized keratinocytes as well as a lipid-laden extracellular matrix (ECM), serves to prevent trans-epidermal water loss. In addition, epidermis is to provide protective shield from external stimuli and to response antigenic epitope as immune sentinels. Moreover, fiber-dense ECM of the dermis protects various histological components (e.g., hair follicles, nerve endings, sweat glands, sebaceous glands, blood and lymphatic vessels) within it and serves mechanical strength and flexibility of skin texture. Furthermore, hypodermal adipose tissue blocks heat gain and loss from the body and protects underlying architectures (e.g., muscles, bones, and internal organs) [1]. In regards, repetitive and chronic damage to skin tissue not only deteriorates skin function, but can also worsen disease status of entire body due to collapse of the first line of physiological and immunological defense barrier.

Among various stimuli, ultraviolet (UV) rays are the most potent extrinsic factor to cause skin damage known as photoaging [2]. UVC (100–280 nm) spectrum is almost completely filtered by the atmospheric gases, but UVA (315–400 nm) and small amounts of UVB (280–315 nm) can reach the Earth’s surface in sufficient quantities to damage skin structures. The amount of UVB reaching the ground has been gradually increasing due to ozone layer depletion [2,3], while the ozone layer has been recovering since the Montreal protocol in 1987. 

Chromophores in epidermis and dermis absorb radiation energy from both UVA and UVB rays, transfer to an excited energy state, and thereby accelerate reactive oxygen species (ROS) production [2,4]. Albeit transiently generated ROS are efficiently removed by the antioxidant defense system (e.g., glutathione, coenzyme Q10, vitamin E, vitamin C, superoxide dismutase, catalase, and glutathione reductase) predisposed in skin tissue, repeated exposure to UV beyond the defense capacity causes oxidative stress and accumulates damaged macromolecules (e.g., DNAs and proteins), which in turn accelerates skin photoaging, including epidermal thickening, wrinkle formation, loss of skin elasticity, dryness, and irregular pigmentation [1]. UVA rays are involved in ROS formation and photoaging. And, the effects of UVB irradiation cause more severe damage to the skin, since UVB has 1000 times stronger irradiation energy than UVA [5]. For instance, UVB can trigger skin erythema and cancer more prevalently than UVA. On the contrary, repeated exposure of a low dose of UVB can also cause skin photoaging. There is accumulated evidence that UVB generates ROS and induces photoaging [5,6,7,8,9].

Unless UV-mediated photoaging is appropriately managed, the pathological consequences of photoaging can aggravate skin damage with hyperpigmented lesions, verrucous papules, telangiectasia, and even skin cancer [2]. To protect the skin from photoaging caused by UV irradiation, it has been recommended to use organic or inorganic sunscreens as well as wear clothing. However, the use of sunscreens has been limited due to the following issues: inconvenience to apply repeatedly for lasting sun protection, irritation in oily or acne-prone skin, cloth staining, immunotoxicological side effects of specific ingredients, and environmental pollution. Therefore, functional foods and their derived phytochemicals with potent antioxidant activity without serious side effects have been receiving great attention [6].

*Elaeagnus umbellata* Thunb., which is generally known as autumn olive or cardinal olive, is found in temperate and subtropical regions of East Asia, Central Asia, South Europe, and North America. In traditional folk medicine, various parts of *E. umbellata* have been used to cure diverse diseases including cardiac disease, respiratory diseases, pulmonary infections, cough, and bowel disorders [10]. Especially, *E. umbellata* fruit, a traditionally edible food stuff, is rich in diverse phytochemicals associated with potent antioxidant activity [11,12,13]. It has been recently proved that *E. umbellata* fruit also has the potential to manage type 2 diabetes, amnesia, and periodontitis [10,12,14]. Despite its strong antioxidant activity, little is known about whether *E. umbellata* fruit can protect the skin from harmful UV rays. 

Previously, we investigated the severity of skin lesions according to the amount of UV radiation, and established that 330 mJ/cm^2^ of UV is an optimal energy to cause skin photoaging without photocarcinogenesis [15,16,17]. Using this model, we made a preliminary comparison of the efficacy of several medicinal plants traditionally reported to have skin protective effects, and found that 200 mg/kg of *E. umbellata* fruit extract is the most potent skin protective candidate. Therefore, the present study investigated the dose-dependent skin protective efficacy of *E. umbellata* fruit up to 200 mg/kg using UV-irradiated hairless mice and compared its effectiveness with ascorbic acid (AA), a well-known antioxidant as well as skin protectant.

## 2. Materials and Methods

### 2.1. Preparation of Elaea and Quantification of Ellagic Acid

To prepare Elaea, uncrushed domestic *E. umbellata* fruits were boiled twice with water, filtered through a 50 μm mesh, concentrated to approximately 26 brix, and spray dried with the addition of 50% (*w*/*w*) dextrin. Elaea dissolved in 80% methanol (0.04 g/mL) was injected into Alliance e2695/2998 high performance liquid chromatography (HPLC) system (Waters; Milford, MA, USA) equipped with CAPCELL PAK C18 UG120 column (column size, 4.6 × 250 mm; pore size, 5 μm) (Osaka soda; Osaka, Japan) and photodiode array detector (Waters, Milford, MA, USA). The mobile phase was mixture of 0.05% trifluoroacetic acid (solution A) and 100% acetonitrile (solution B). Elaea was eluted in the following gradient mode: 16% solution B over 0 min, 19% solution B over 30 min, 95% solution B over 31 min, and 95% solution B over 40 min. The eluants were detected at a wavelength of 254 nm. The content of ellagic acid in Elaea was quantified by comparing area of the peak showing the same retention time with that of the standard compound. 

### 2.2. Measurement of Total Flavonoids Content

Total flavonoids content of Elaea was quantified according to a previously established method with minor modifications [18]. Briefly, 100 μL of Elaea (10 mg/mL) or serially diluted quercetin (Sigma-Aldrich; St. Louis, MO, USA) was reacted with 20 μL of 10% aluminum nitrate and 20 μL of 1 M potassium acetate in 860 μL of dimethyl sulfoxide for 40 min at room temperature. Optical intensity was measured at a wavelength of 415 nm using an Enspire™ microplate reader (PerkinElmer; Waltham, MA, USA).

### 2.3. Animal Experiment and Treatment

Protocol for animal experiment was reviewed and approved from Institutional Animal Care and Use Committee of Daegu Haany University (Approval No. DHU2022-059; Approval date, 23 June 2022). Sixty six CrlOri:SKH1-hr hairless mice (gender, female; age, 6 weeks old; body weight, 20–24 g) were supplied from OrientBio (Seungnam, Republic of Korea) and maintained under standard breeding conditions (e.g., temperature, 20–25 °C; humidity, 50–55%; 12 h:12 h light and dark cycle; unrestricted access to food and water). After one week of acclimatization, six mice were excluded due to uneven body weight distribution, and sixty mice were randomly assigned to 6 experimental groups (*n* = 10/group): Control, UV, UV + AA, UV + Elaea (200), UV + Elaea (100), and UV + Elaea (50). Using a CL-1000M UV crosslinker (Analytik Jena; Upland, CA, USA) equipped with five Hg UV lamps (peaked emission wavelength, 312.8 nm), mice were exposed to 330 mJ/cm^2^ of UV, consisting of 137 mJ/cm^2^ UVA, 190 mJ/cm^2^ of UVB, and 3 mJ/cm^2^ of UVC, three times per weeks for 15 weeks. A total of 50–200 mg/kg of Elaea or 100 mg/kg AA dissolved in distilled water was orally administered to mice once daily for 15 weeks. On the day of UV irradiation, each drug was administered 1 h after UV exposure. Control mice stayed in the chamber for the same amount of time without UV irradiation (approximately 55 s), and mice in the control and UV groups received the same volume of distilled water instead of the test article. All mice were euthanized 24 h after the last drug administration.

### 2.4. Body Weight and Skin Weight Measurement

On days 0 and 105 (i.e., day 0 = the first day of drug administration), all animals were fasted chow for approximately 18 h to reduce the individual body weight differences through heterogeneous feeding. Using an XB320M automatic balance (Precisa instrument; Zürich, Switzerland), the body weight was measured once a week from 1 day before the first drug administration until the end of administration. In addition, dorsal skins of euthanized mice were collected from the epidermis to the subcutaneous layer. Skin weight and water contents were measured after punching a 6 mm diameter piece of skin tissue.

### 2.5. Histopathology and Immunohistochemistry

Preparation of tissue sections from dorsal skin around gluteal regions, hematoxylin and eosin staining for general histopathological assessment, and immunohistochemistry were conducted, as previously described [15,16]. After observing tissue sections under light microscope (Eclipse 80*i*, Nikon; Tokyo, Japan) equipped with a ProgRes C5 histological camera (Jenoptik Optical Systems GmbH; Jena, Germany) and an image analyzer (*i*Solution FL 9.1, IMT *i*-solution; Burnaby, BC, Canada), the number of microfolds per mm epidermal surface, thickness of the epithelial layer excluding the basement membrane of the epidermis (μm), and the number of infiltrated inflammatory cells per mm^2^ of dermis were counted from the hematoxylin and eosin-stained skin sections. For immunohistochemistry, specific antibody directed cleaved caspase-3 (Cell signaling technology; Beverly, MA, USA), cleaved poly ADP-ribose polymerase (PARP) (Santa Cruz Biotechnology; Santa Cruz, CA, USA), matrix metalloproteinase (MMP)-9 (Abcam; Cambridge, UK), nitrotyrosine (Millipore; Temecula, CA, USA), or 4-hydroxynonenal (Abcam) was used for reacting with skin sections. Cells showing an increase in intensity of 40% or more compared to the background were considered immunoreactive cells. Using an image analyzer (*i*Solution FL 9.1), the number of cleaved caspase-3-, cleaved PARP-, nitrotyrosine-, or 4-hydroxynonenal-immunoreactive cells was counted per 100 epidermal cells. For MMP-9, immunoreactive fibers in the dermis were calculated as a percentage per mm^2^ of dermis.

### 2.6. Skin Replica Analysis

After a photograph of the dorsal skin around gluteal region was taken using a digital camera (FinePix S700, Fujifilm; Tokyo, Japan), replicas of the dorsal skin of mice were generated using a Repliflo cartridge kit (CuDerm Co.; Dallas, TX, USA). The wrinkle shadow of skin replica was produced by illuminating with fixed intensity light at a 40° angle using an optical light source, and then the mean length (mm) and depth (μm) of wrinkles in the replica were analyzed using a SV600 Skin-Visiometer System (Courage & Khazaka; Cologne, Germany).

### 2.7. Enzyme-Linked Immunosorbent Assay (ELISA)

Skin tissues in radioimmunoassay precipitation buffer were homogenized by using a Taco™ Prep bead beater (GeneReach Biotechnology; Taishung, Taiwan) and a KS-750 ultrasonic disruptor (Madell Technology; Ontario, CA, USA), centrifuged at 15,000× *g* for 10 min, and then supernatants were collected as skin homogenates. According to the manufacturer’s instructions, levels of type I collagen, hyaluronan, interleukin (IL)-1β, and IL-10 in skin homogenates were measured using procollagen type I C-peptide (PIP) EIA kit (Takara; Shiga, Japan), mouse hyaluronic acid ELISA Kit (Mybiosource; San Diego, CA, USA), mouse IL-1β ELISA Kit (Abcam), and Mouse IL-10 ELISA Kit (Abcam), respectively. In case of hyaluronan measurement, trichloroacetic acid was added and then centrifuged to remove proteins from the skin homogenates.

### 2.8. Measurement of Water Content

Water content (%) in skin tissue punched with diameter of 6 mm was measured using MB23 moisture balance (OHAUS; Parsippany, NJ, USA).

### 2.9. Quantitative Polymerase Chain Reaction (qPCR)

Total RNA isolation from skin tissue, RNA quantification, reverse transcription, and qPCR were carried out, as previously described [15]. Specific primer pairs for amplifying murine *mmp-9*, *mmp-1*, *mmp-13*, *p38 mitogen-activated protein kinase* (*mapk*), *glutathione reductase* (*gsr*), *NADPH oxidoreductase 2* (*nox2*), and *β-actin* obtained from OriGene Technologies (Rockville, MD, USA) were listed in Table 1. Relative expression level of specific gene was calculated by the ΔΔC_T_ method [19] using *β-actin* as a housekeeping gene.

### 2.10. Measurement of Reduced Glutathione

Proteins in skin homogenate were removed by centrifugation after addition of 30% trichloroacetic acid. Resulting supernatant was reacted with *o*-phthalaldehyde in 0.1 M sodium phosphate buffer (pH 8.0) for 15 min, and fluorescence intensity was monitored at an excitation wavelength of 350 nm and an emission wavelength of 420 nm using a RF-5301PC fluorescence spectrophotometer (Shimadzu; Tokyo, Japan). Glutathione level of skin homogenates was normalized to protein concentration. 

### 2.11. Measurement of Lipid Peroxidation

Deproteinated skin homogenates were reacted with 0.67% thiobarbituric acid at 100 °C for 15 min. Malondialdehyde level was determined by difference in absorbance between 535 and 572 nm, and normalized to protein concentration.

### 2.12. Measurement of Superoxide Anion Production

Superoxide anion production in the skin was quantified by nitroblue tetrazolium assay. Briefly, skin homogenates were incubated with nitroblue tetrazolium chloride at 37 °C for 1 h, and clarified by centrifugation. The formazan crystals in the resulting pellets were solubilized by adding 2M potassium hydroxide in dimethyl sulfoxide. Optical intensity was monitored at 620 nm of wavelength, and normalized to protein concentration.

### 2.13. Measurement of Myeloperoxidase Activity

In 50 mM potassium phosphate buffer (pH 6.0) containing 0.05% hexadecryltrimethylammonium bromide, the skin tissue was disrupted with a bead beater and an ultrasonicator, and then clarified by centrifugation. After homogenates were incubated with 50 mM phosphate buffer (pH 6.0) containing 0.167 mg/mL *o*-dianisidine dihydrochloride and 0.05% hydrogen peroxide for 5 min, optical intensity at 450 nm was measured using an Optizen POP spectrophotometer (Mecasys; Daejeon, Republic of Korea). Myeloperoxidase activity of skin homogenates was calculated by interpolating a standard curve of neutrophils and was normalized to protein concentration.

### 2.14. Statistical Analysis

All numerical results are expressed as means ± standard deviations and analyzed using a SPSS Statistics 18 software (SPSS Inc.; Chicago, IL, USA). Based on the results of Levene’s test, one-way analysis of variance or Welch’s test was performed to compare means between experimental groups, followed by Tukey’s honestly significant difference test or Dunnett’s T3 test as post hoc analysis. A *p*-value less than 0.05 was considered a significant difference.

## 3. Results

### 3.1. Elaea Prevents Apoptosis in the Skin of UV-Irradiated Mice

Before investigating the beneficial effects of Elaea in UV-mediated skin photoaging, we quantified the total flavonoids and ellagic acid contents of Elaea. Colorimetric detection showed that Elaea used in this study contained 655.86 ± 64.30 quercetin equivalent μg/g of total flavonoids. 

**Figure 1:**
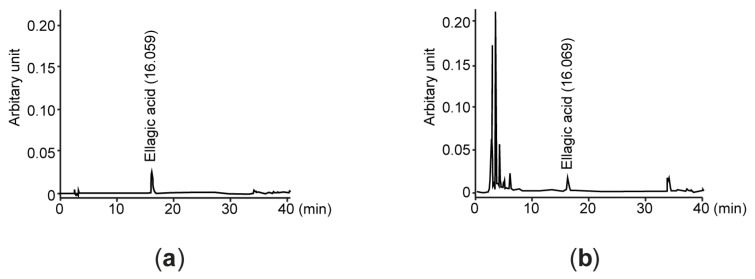
HPLC chromatogram of Elaea. Representative HPLC chromatograms were detected at 254 nm of wavelength after eluting ellagic acid (**a**) and Elaea (**b**). Numbers in each chromatogram are retention times of ellagic acid; Elaea—*E. umbellata* fruit extract; HPLC—high-performance liquid chromatography.

In addition, result from HPLC analysis revealed that Elaea had 83.93 ± 0.32 μg/g of ellagic acid (Figure 1).

**Figure 2:**
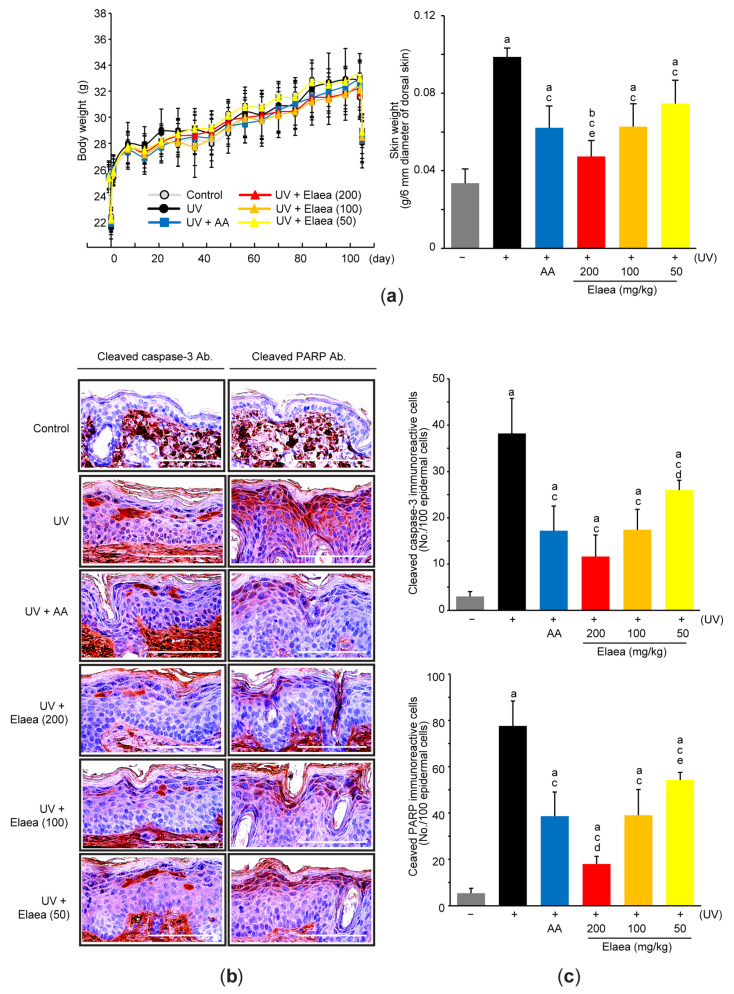
Elaea prevents skin epidermal apoptosis in UV-irradiated mice. (**a**) Body weight was measured during the entire experimental period (**left**), and skin weight with a diameter of 6 mm was determined from euthanized mice at the end of the experiment (**right**). (**b**) Representative immunohistochemical images from the skin sections which were reacted with antibodies against cleaved caspase-3 (**left**) or cleaved PARP (**right**). Scale bars indicate 100 μm. (**c**) Cleaved caspase-3- (**upper**) and cleaved PARP-immunoreactive cells (**lower**) in the epidermis were counted using an image analyzer. ^a^ *p* < 0.01, ^b^ *p* < 0.05 versus control group; ^c^ *p* < 0.01 versus UV group; ^d^ *p* < 0.01, ^e^ *p* < 0.05 versus UV + AA group; AA—ascorbic acid; Ab.—antibody; PARP—poly ADP ribose polymerase; UV—ultraviolet.

Next, 50–200 mg/kg of Elaea or 100 mg/kg of AA was orally administered into hairless mice once a day for 15 weeks, and UV (330 mJ/cm^2^) was irradiated three times a week for 15 weeks to induce skin photoaging. When body weights of all mice were measured once a week, there was no difference in body weight between groups of mice during the entire experimental period (Figure 2a-left). However, after the experiment, the weight of dorsal skin around the gluteal regions with a diameter of 6 mm was significantly increased in the UV-irradiated mice group compared to the control mice. Oral administration of three different doses of Elaea or AA significantly reduced UV-mediated skin weight gain (Figure 2a-right). To explore the effect of Elaea on UV-induced skin injury, skin sections were stained with specific antibodies correlated with apoptosis. Our immunohistochemical results revealed that UV irradiation significantly increased the number of cleaved caspase-3- (Figure 2b-left,c-upper) and cleaved PARP-immunoreactive cells (Figure 2b-right,c-lower) in the skin epidermis, suggesting that repeated exposure to UV activates epidermal apoptosis. However, UV-mediated apoptosis induction was decreased in mice given 50–200 mg/kg Elaea (Figure 2b,c). The effects of 100 mg/kg Elaea administration on reducing skin weight and the number of cleaved caspase-3- and cleaved PARP-immunoreactive cells was similar to that of AA administration, but the reduction in skin weight and cleaved PARP-immunoreactive cells by administration of 200 mg/kg Elaea greater than that by AA (Figure 2).

### 3.2. Elaea Reduces Wrinkle Formation in the Skin of UV-Irradiated Mice

**Figure 3:**
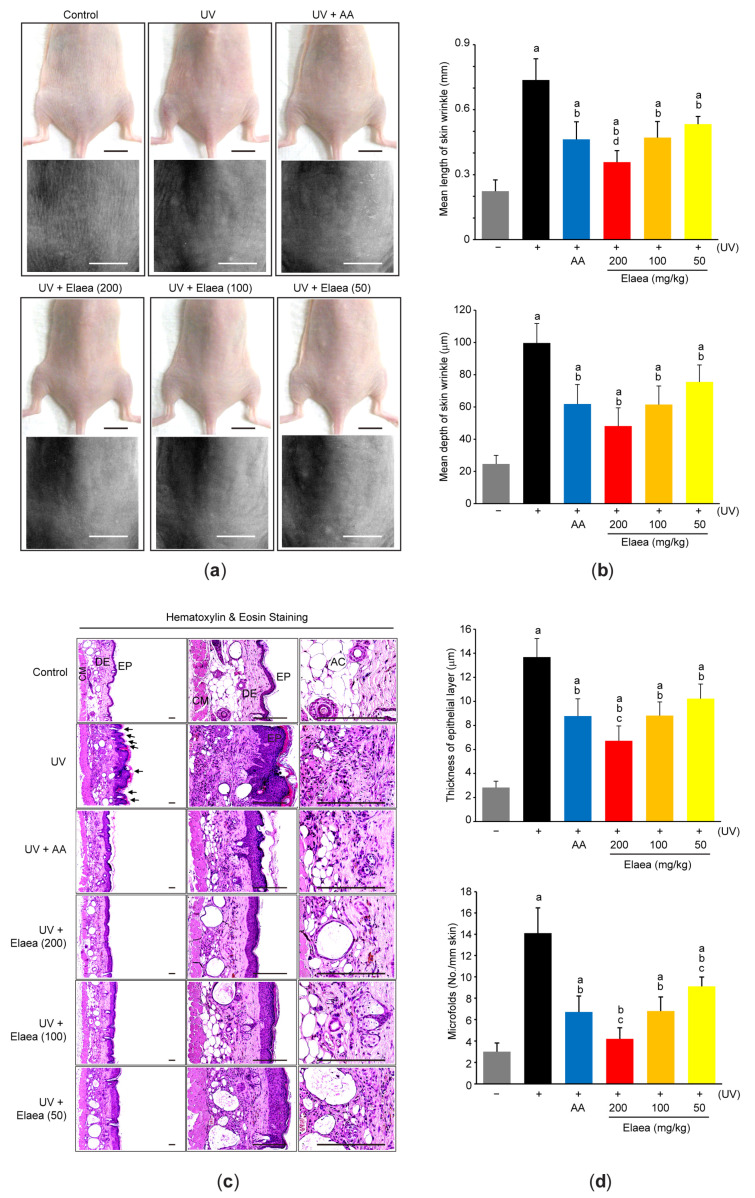
Elaea reduces wrinkle formation in the skin of UV-irradiated mice. (**a**) Representative images of dorsal skin (**upper**) and replica (**lower**). Scale bars indicate 10 mm. (**b**) The wrinkle length (**upper**) and wrinkle depth (**lower**) in skin replicas were measured using a skin-visiometer system. (**c**) Representative histological images after hematoxylin and eosin staining of skin tissue section. Scale bars indicate 200 μm, and arrows indicate microfolds formed on the surface of epidermis. (**d**) Thickness of the epithelial layer in the epidermis (**upper**) and the number of microfolds (**lower**) were measured using an automated image analyzer. ^a^ *p* < 0.01 versus control group; ^b^ *p* < 0.01 versus UV group; ^c^ *p* < 0.01, ^d^ *p* < 0.05 versus UV + AA group; AC—adipocyte; CM—cutaneous muscle; DE—dermis; EP—epidermis.

To explore the effect of Elaea on UV-induced skin wrinkle, skin replicas were generated from the dorsal skin around gluteal region (Figure 3a). When macroscopic lesions were observed using a skin-visiometer system, the length (Figure 3b-upper) and depth (Figure 3b-lower) of skin wrinkles were increased in the replicas of UV-irradiated mice. However, administration of three different doses of Elaea significantly alleviated the UV-mediated wrinkle formation (Figure 3a,b). Next, skin sections were stained with hematoxylin and eosin to further observe the effect of Elaea administration on UV-induced microscopic skin lesions (Figure 3c). Repeated UV exposure caused an increase in the thickness of the epithelial layer due to abnormal hyperplasia (/hypertrophy) of epidermal keratinocytes (Figure 3c,d-upper). Moreover, UV irradiation increased microfolds on the epithelial surface (Figure 3c,d-lower). However, these UV-induced histopathological changes were dose-dependently mitigated in mice skin administered with 50–200 mg/kg Elaea (Figure 3c,d). Except for wrinkle depth, reductions in macroscopic and microscopic wrinkles in mice administered with 200 mg/kg Elaea were greater than those due to AA administration, but the extent of wrinkle reduction by 100 mg/kg Elaea administration was not different from AA (Figure 3b,d).

### 3.3. Elaea Inhibits ECM Degradation in the Skin of UV-Irradiated Mice

**Figure 4:**
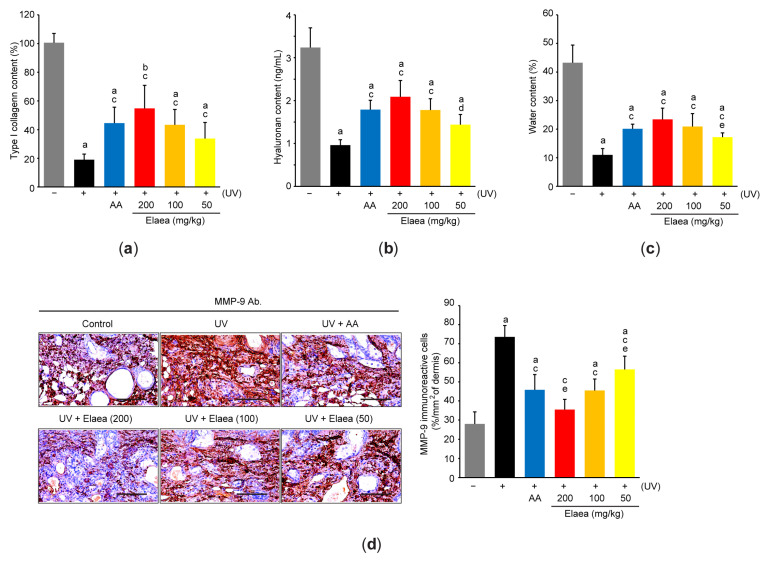
Elaea inhibits ECM degradation in the skin of UV-irradiated mice. The content of type I collagen (**a**) and hyaluronan (**b**) in skin homogenate was determined using commercial ELISA kits. (**c**) The water content of skin tissue having a diameter of 6 mm was measured using a moisture analyzer. (**d**) Representative immunohistochemical images obtained after incubation of skin sections with an MMP-9 antibody. Scale bars indicate 100 μm (**left**). The MMP-9-immunoreactive area of the dermis was counted using an image analyzer (**right**). ^a^ *p* < 0.01, ^b^ *p* < 0.05 versus control group; ^c^ *p* < 0.01, ^d^ *p* < 0.05 versus UV group; ^e^ *p* < 0.01 versus UV + AA group; ELISA—enzyme-linked immunosorbent assay; MMP-9—matrix metalloproteinase-9.

To explore the effect of Elaea on ECM in skin, we measured the level of type I collagen and hyaluronan which are representative components in skin ECM [1]. As expected, ELISA analysis using skin homogenates showed that UV significantly decreased the type I collagen and hyaluronan levels. However, administration of three different doses of Elaea significantly prevented the reduction in the ECM components (Figure 4a,b). In addition, UV-mediated water depletion in skin tissue was also significantly blocked in mice administered with three different doses of Elaea (Figure 4c). Consistently, immunohistochemical and real-time qPCR analyses showed increased expression levels of key ECM degrading enzymes such as *mmp-9*, *-1,* and *-13* in skin tissues from UV-irradiated mice. Moreover, mRNA level of *p38 mapk*, an upstream signaling molecule to activate *mmp*s transcription [20], also increased in UV-exposed skin (Table 2). However, UV-mediated induction of *mmp*s and *p38 mapk* in skin tissue was inhibited in mice given three different doses of Elaea (Figure 4d and Table 2). Inhibition of ECM loss, blockage of water depletion, and reduction in *mmp-9*/*mmp-13* mRNA expression in skin tissue by 100 and 200 mg/kg Elaea administration were similar to those by AA, but the effect of 200 mg/kg Elaea administration on the number of MMP-9 immunoreactive cells, *mmp-1* and *p38 mapk* mRNA level in mice skin was greater than that of AA administration (Figure 4 and Table 2).

### 3.4. Elaea Alleviates Oxidative Stress in the Skin of UV-Irradiated Mice

**Figure 5:**
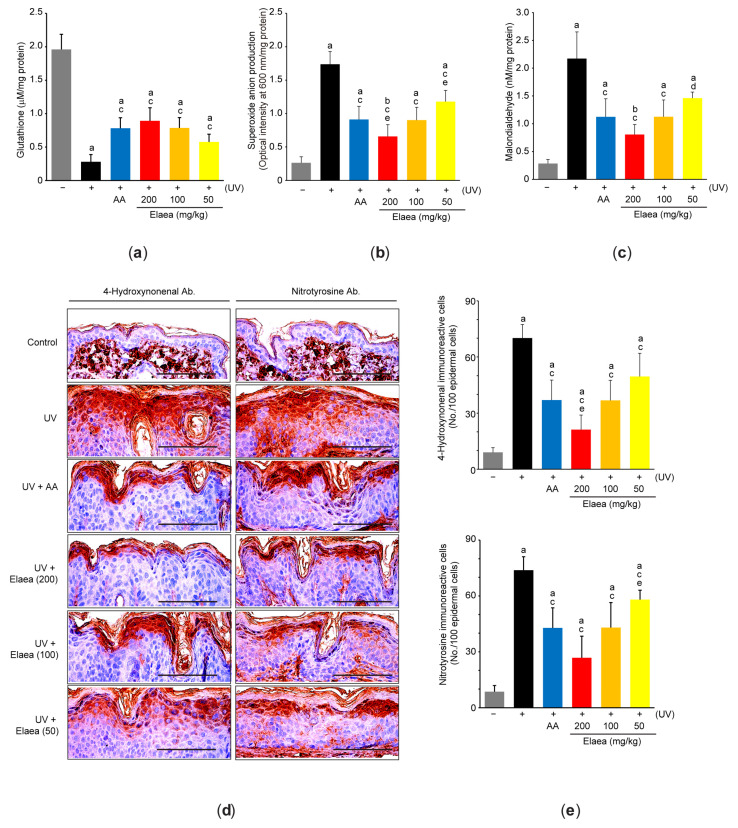
Elaea alleviates oxidative stress in the skin of UV-irradiated mice. Contents of glutathione (**a**), superoxide anion (**b**), and malondialdehyde (**c**) in skin homogenate was measured, as described in Materials and Methods. (**d**) Representative immunohistochemical images after incubating skin sections with 4-hydroxynonenal (**left**) or nitrotyrosine (**right**) antibody. Scale bars indicate 100 μm. (**e**) 4-Hydroxynonenal- (**upper**) and nitrotyrosine-immunoreactive cells (**lower**) in the epidermis were counted using an image analyzer. ^a^ *p* < 0.01, ^b^ *p* < 0.05 versus control group; ^c^ *p* < 0.01, ^d^ *p* < 0.05 versus UV group; ^e^ *p* < 0.05 versus UV + AA group.

To understand mechanisms associated with Elaea-mediated skin protection, we measured changes in representative markers of oxidative stress in skin tissue. As expected, levels of glutathione (Figure 5a) and *gsr* mRNA (Table 2), essential for maintaining the reducing environment of cells and resisting oxidative stress [21], were significantly reduced in the skin of UV-irradiated mice. In contrast, *nox2* mRNA (Table 2), superoxide anion (Figure 5b), lipid peroxidation [e.g., malondialdehyde (Figure 5c), and 4-hydoxynonenal-immunoreactive cells (Figure 5d-left,e-upper)], and protein nitration (e.g., nitrotyrosine-immunoreactive cells) (Figure 5d-right,e-lower) in skin were significantly increased by UV irradiation. However, administration with three different doses of Elaea was significantly alleviated UV-mediated depletion of antioxidant defense system and accumulation of oxidative byproducts in the skin (Figure 5a–e and Table 2). Alleviation of skin oxidative stress by 100 and 200 mg/kg Elaea was not statistically differed from the effect of AA administration, except for superoxide anion production and 4-hydroxynonenal immunoreactive cells between UV + 200 mg/kg Elaea and UV + AA (Figure 5a–e and Table 2).

### 3.5. Elaea Decreases Inflammatory Response in the Skin of UV-Irradiated Mice

**Figure 6:**
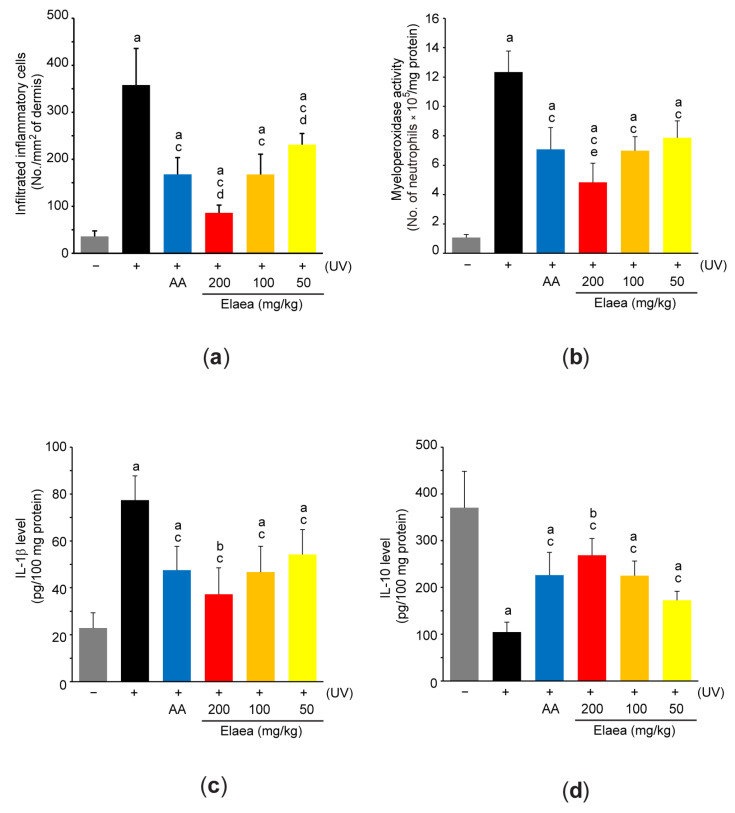
Elaea decreases inflammation in the skin of UV-irradiated mice. (**a**) The number of infiltrated inflammatory cells in the hematoxylin and eosin-stained skin section was counted under a light microscope equipped with an automated image analyzer. (**b**) Myeloperoxidase activity of skin homogenate was measured by H_2_O_2_-dependent oxidation of *o*-dianisidine. The IL-1β (**c**) and IL-10 (**d**) contents in skin homogenate was determined using commercial ELISA kits. ^a^ *p* < 0.01, ^b^ *p* < 0.05 versus control group; ^c^ *p* < 0.01 versus UV group; ^d^ *p* < 0.01, ^e^ *p* < 0.05 versus UV + AA group; IL—interleukin.

Because it has been reported that UV irradiation destroys skin architecture along with activation of inflammation [1], we further tested the possibility that Elaea protects the skin from UV exposure via regulating inflammation. For this, we counted the number of infiltrated inflammatory cells in hematoxylin and eosin-stained skin tissue and found that UV significantly increased the number of inflammatory cells in skin section (Figure 3c and Figure 6a). Compared to control, skin homogenates of UV-irradiated mice were also significantly increased *o*-dianisidine oxidation in the presence of H_2_O_2_ (Figure 6b), which was primarily resulted from myeloperoxidase in infiltrating neutrophils [22]. However, the number of infiltrated inflammatory cells as well as the myeloperoxidase activity in skin tissue were significantly reduced in groups of mice administered with three different doses of Elaea. Moreover, IL-1β (a proinflammatory cytokine) and IL-10 (an anti-inflammatory cytokine), which had been reciprocally regulated in the skin of UV-irradiated mice, were also restored toward basal levels by the administration of three different doses of Elaea (Figure 6c,d). Inhibition of inflammatory cell infiltration including neutrophils and recovery of inflammation-related cytokines by 100 and 200 mg/kg Elaea administration were similar to those by AA administration, except for the number infiltrated inflammatory cells and myeloperoxidase activity between UV + 200 mg/kg Elaea and UV + AA (Figure 6a–d).

## 4. Discussion

Diverse phytochemicals such as polyphenols (e.g., ellagic acid, chlorogenic acid, gallic acid, cinnamic acid, and benzoic acid), flavonoids (e.g., epigallocatechin gallate, myricetin, and morin), terpenoids (e.g., lycopene, β-carotene, lutein, phytoene, and cryptoxanthin), vitamins (e.g., vitamin A, C, and E), and fatty acids (e.g., linoleic acid) have been identified from *E. umbellata* fruit extract [12,13,14,23]. Among them, it has been reported that lycopene in *E. umbellata* fruit is 5 to 20 times more abundant than in tomato [24]. Moreover, topical application as well as oral ingestion of lycopene has been known to reduce skin erythema in UV-exposed volunteers [25,26]. However, our preliminary analysis revealed that lycopene in Elaea was present below the limit of detection. Because Elaea has been prepared by extracting *E. umbellata* fruit in hydrophilic condition, it is possible that water-insoluble phytochemicals including lycopene are rarely extracted in Elaea. On the contrary, Elaea used in this study was found to contain 655.86 ± 64.30 quercetin equivalent μg/g of flavonoids and 83.93 ± 0.32 μg/g of ellagic acid. In an effort to identify valuable phytochemicals in Elaea, Elaea was further analyzed by using an ultraperformance liquid chromatography (UPLC)-quadrupole time-of-flight (QTOF)/mass spectrometry (MS). The identified compounds were analyzed based on the library score above 70 acquired from MS/MS fragmentation pattern and precursor mass tolerance ± 0.02 Da. Throughout UPLC-QTOF/MS analysis, we identified at least 54 secondary metabolites (18 compounds in electron spray ionization positive ion mode, 23 compounds in electron spray ionization negative ion, and 13 compounds in both ion mode) in Elaea (Table S1). Interestingly, it has been reported that several phytochemicals identified in Elaea through UPLC-QTOF/MS analysis (e.g., ellagic acid, gallic acid, protocatechuic acid, trigonelline, afzelin, astragalin, rutin, quercetin, glycitin, decursin, morin, myricetin, *p*-coumaric acid, catechin, dihydromyricetin, and polydatin: compounds are ordered by peak area in the UPLC chromatogram) are capable to alleviate ROS production, apoptosis, skin wrinkles, epidermal hyperplasia, and skin inflammation induced by UV irradiation [17,27,28,29,30,31,32,33,34,35,36,37,38,39,40,41]. Therefore, present results suggest that aforementioned phytochemicals identified through UPLC-QTOF/MS analysis together probably contribute to protecting skin damages from repetitive UV exposure.

Direct absorption of UV energy in skin endogenous chromophores (e.g., urocanic acid, riboflavin, bilirubin, melanin, porphyrin, pterins, and heme) facilitates to generate free radicals and singlet oxygens via electron transfer-mediated hydrogen abstraction and energy transfer with oxygen [4,42]. In addition, respiratory burst by NAPDH oxidase (e.g., NOX2) in infiltrated inflammatory cells and keratinocytes rapidly releases ROS in UV-exposed skin [43,44]. Moreover, excessively generated ROS which overwhelm endogenous antioxidant capacity accumulates damaged biomolecules (e.g., oxidized fatty acid and nitrosylated proteins), which ultimately leads cell fate toward death. In the present study, we also showed that repeated UV exposure increased the level of superoxide anion, fatty acid oxidation (e.g., malondialdehyde and 4-hydroxynonenal), protein nitrosylation at tyrosine residue, and *nox2* mRNA. On the contrary, UV decreased glutathione and glutathione reductase, suggesting that UV causes oxidative stress along with depleting antioxidant defense systems. However, oral administration of Elaea for 15 weeks suppressed changes in the aforementioned biomarkers in a dose dependent manner. Therefore, present results provide strong evidence that Elaea can protect skin from UV-mediated photoaging by relieving oxidative stress.

In the present study, we showed chronic exposure to UV increased cleaved caspase-3 and cleaved PARP immunoreactive cells in skin tissue. In addition, present results revealed that UV significantly increased the weight of the punched dorsal skin and the thickness of the epithelial layer. It has been reported that UV-induced ROS provokes p53-dependent cell cycle arrest by accumulating premutagenic lesions (e.g., cyclobutane pyrimidine dimers, pyrimidine-pyrimidone (6-4) photoproducts and Dewar valence isomers) in DNA [6,45]. Moreover, ROS activates the intrinsic signaling pathway of apoptosis through disruption of mitochondrial membrane, cytoplasmic release of mitochondrial cytochrome c and subsequential activation of the apoptosome/caspase-9/caspase-3/PARP axis [46]. Especially, UV-induced apoptosis of skin cells, generally known as sunburn cells, displays distinct morphological characteristics such as pyknotic nuclei with diskeratotic and vacuolated cytoplasm, mitochondrial swelling, and rupture [6,47]. Furthermore, skin damage in response to repeated UV irradiation promotes rapid proliferation of remaining keratinocytes, resulting in thickening of the epithelial layer in the epidermis [6,16]. Therefore, the present results showing Elaea decreased the number of caspase-3/PARP-immunoreactive cells and reduced epithelial hyperplasia in a dose-dependent manner provide evidence that Elaea can suppress epidermal thickening by inhibiting UV-mediated apoptosis in photoaged skin.

In the present study, we showed that administration of three different doses of Elaea could effectively prevent not only histological microfold but also macroscopic wrinkle formation in the skin of UV-irradiated mice. Several mechanisms involving in UV-dependent disorganization of ECM have been reported to form wrinkle in photoaged skin. UV perturbs transforming growth factor β signaling pathway by activating Smad7, an inhibitory Smad, and thereby downregulates collagen synthesis [48]. In response to UV irradiation, dermal fibroblast, epidermal keratinocytes, and infiltrated inflammatory cells (e.g., neutrophils) also induce the transcription of *mmp* family and *elastase* for accelerating ECM degradation [49,50]. MMP-1 (interstitial collagenase), MMP-2 (72 kDa gelatinase), MMP-9 (92 kDa gelatinase), and MMP-13 (collagenase 3) in epidermis have been known as representative MMPs induced by UV exposure [49,51]. Moreover, UV has been reported to decrease the level of dermal hyaluronan by down-regulating hyaluronic acid synthase and inhibiting transforming growth factor β signaling pathway [52]. Furthermore, reduction in fibrillin structures and type VII collagen also contribute to forming fragile interface at dermal-epidermal junction [53]. In the present study, we showed that Elaea administration inhibited the UV-mediated decrease in type I collagen and hyaluronan. In addition, Elaea also decreased the induction of *mmp* mRNAs in UV-exposed skin. Therefore, present results suggest that Elaea reduces wrinkle formation by restoring ECM components and inhibiting ECM degrading enzymes.

Sunburned keratinocytes directly secrete inflammatory mediators (e.g., cytokines and prostanoids) by activation of ROS-dependent signaling pathway [1,54]. In addition, various damage-associated molecular patterns generated by resident cells in UV-exposed skin are known to trigger the infiltration of phagocytic inflammatory cells through dermal lymphatic vessels [55]. Our histopathological analysis against hematoxylin and eosin-stained skin also confirmed UV increased the number of dermal inflammatory cells. In addition, the increase in skin myeloperoxidase activity by UV suggests that the infiltrated cells in the dermis are probably proinflammatory cells, including neutrophils. Moreover, the increase in proinflammatory cytokine (e.g., IL-1β) and decrease in anti-inflammatory cytokine (e.g., IL-10) in tissue homogenates further support the concept that UV irradiation activates chronic low-grade inflammation in skin tissue. Therefore, our results by Elaea administration suggest that Elaea can protect the skin through managing acute inflammation.

One of the best-studied cellular responses against to UV is the activation of the MAPK signaling pathway [56], and our results obtained from qPCR analysis also confirmed that UV exposure increased mRNA level of *p38 mapk* in skin tissue. It has been reported that chemical inhibition of p38 MAPK suppressed the sunburn cell formation, apoptosis, and inflammation in UV-irradiated animals [47]. In addition, p38 MAPK in UV exposed keratinocytes initiates apoptosis by promoting mitochondrial translocation of cytoplasmic Bax [57,58]. Moreover, UV-mediated p38 activation also inhibits cell cycle progression through modulating p53, p21, cdc25, and 14-3-3 [59,60,61]. Furthermore, UV-dependent p38 MAPK activation subsequently increases c-fos expression and transactivates activator protein-1, which in turn induces proinflammatory cytokines and MMPs expression [20,62,63,64]. Therefore, inhibition of *p38 mapk* mRNA by Elaea administration may be one of the putative mechanisms by which Elaea protects the skin from UV irradiation via reducing premature senescence, apoptosis, ECM remodeling, oxidative stress, and inflammation. Although Elaea was shown to suppress *p38 mapk* mRNA in this study, p38 MAPK activity is known to be also regulated by post-translational modification (e.g., phosphorylation) [59]. So, the effect of Elaea on protein modification of p38 MAPK, as well as the effect of Elaea on other MAPKs, needs to be further elucidated in the future.

## 5. Conclusions

Present results clearly showed that oral administration of 50–200 mg/kg *E. umbellata* fruit extract (Elaea) for 15 weeks could prevent epidermal thickening, apoptosis, and wrinkle formation by reducing oxidative stress and infiltration of inflammatory cells in skin repeatedly exposed to UV. In addition, the photoaging prevention effect of 100 mg/kg Elaea was statistically similar to that of 100 mg/kg ascorbic acid, at least for all biomarkers observed in this study. Nevertheless, present study has several limitations worth mentioning: (1) The UV lamp used in the present study emits 42% of UVA, 57% of UVB, and 1% of UVC. Although a number of factors (e.g., variation in stratospheric ozone layer, geographical latitude and altitude, season, time of day, clouds, surface reflection, and air pollution) affect the level of terrestrial UV radiation, the average of daily integrated UVA and UVB irradiance in Busan (35.23° N, 129.07° E; city on the Korean Peninsula) from September 1997 to February 1999 was reported to be 96,800 ± 51,200 and 2120 ± 1510 mJ/cm^2^ [65]. This suggests that the amount and proportion of specific UV ray emitted by artificial UV lamp are quite different from the UV rays emitted by sunlight. Therefore, additional study is needed on the effects of Elaea in UV irradiation models with similar proportions to sunlight. (2) Other biomarkers (e.g., premutagenic DNA lesions including cyclobutane pyrimidine dimers, MMPs proteins levels and those activities) altered by photoaging need to be further explored to solidify and expand the beneficial effects of Elaea. (3) Among the various phytochemicals identified through UPLC-QTOF/MS analysis of Elaea, the active compounds that are correlated with Elaea-mediated photoprotection of skin tissue need to be further elucidated. (4) Finally, efficacy and safety of Elaea should be evaluated in randomized clinical trial in human volunteers. If several remaining issues are appropriately addressed in the future, 100–200 mg/kg of *E. umbellata* fruit (human equivalent dose of 500–1000 mg/day) would be a promising nutraceutical for preventing photoaging and maintaining homeostasis of skin.

## Figures and Tables

**Table 1:** Oligonucleotide sequences used in the present study.

Gene Name	Direction	Nucleotide Sequence	RefSeq Number	Amplicon Size (bp)
*mmp-9*	Forward Backward	5′-GCTGACTACGATAAGGACGGCA-3′ 5′-TAGTGGTGCAGGCAGAGTAGGA-3′	NM_013599.5	136
*mmp-1*	Forward Backward	5′-AGGAAGGCGATATTGTGCTCTCC-3′ 5′-TGGCTGGAAAGTGTGAGCAAGC-3′	NM_032006.3	98
*mmp-13*	Forward Backward	5′-GATGACCTGTCTGAGGAAGACC-3′ 5′-GCATTTCTCGGAGCCTGTCAAC-3′	NM_008607.2	130
*p38 mapk*	Forward Backward	5′-CCGAACGATACCAGAACCTGTC-3′ 5′-ACGCAACTCTCGGTAGGTCCTT-3′	NM_011951.3	158
*gsr*	Forward Backward	5′-GTTTACCGCTCCACACATCCTG-3′ 5′-GCTGAAAGAAGCCATCACTGGTG-3′	NM_010344.4	110
*nox2*	Forward Backward	5′-TGGCGATCTCAGCAAAAGGTGG-3′ 5′-GTACTGTCCCACCTCCATCTTG-3′	NM_007807.5	108
*β* *-actin*	Forward Backward	5′-CATTGCTGACAGGATGCAGAAGG-3′ 5′-TGCTGGAAGGTGGACAGTGAGG-3′	NM_007393.5	138

*mmp*—*matrix metalloproteinase*; *mapk*—*mitogen-activated protein kinase*; *gsr*—*glutathione reductase*; *nox2*—*NADPH oxidoreductase 2*.

**Table 2:** Effect of Elaea on mRNA expression of specific genes associated with UV-mediated photoaging.

Group	Gene Name					
	*mmp-9*	*mmp-1*	*mmp-13*	*p38 mapk*	*gsr*	*nox2*
Control	1.00 ± 0.07	1.00 ± 0.07	1.00 ± 0.05	1.00 ± 0.06	1.00 ± 0.06	1.00 ± 0.05
UV	3.06 ± 0.44 ^a^	2.88 ± 0.20 ^a^	3.72 ± 0.81 ^a^	5.23 ± 0.81 ^a^	0.26 ± 0.06 ^a^	4.09 ± 0.44 ^a^
UV + AA	1.90 ± 0.28 ^a,b^	1.89 ± 0.29 ^a,b^	2.34 ± 0.38 ^a,b^	3.43 ± 0.58 ^a,b^	0.55 ± 0.11 ^a,b^	2.57 ± 0.61 ^a,b^
UV + Elaea (200)	1.54 ± 0.19 ^a,b^	1.45 ± 0.14 ^a,b,e^	1.86 ± 0.29 ^a,b^	2.03 ± 0.38 ^a,b,d^	0.62 ± 0.14 ^a,b^	2.05 ± 0.24 ^a,b^
UV + Elaea (100)	1.89 ± 0.26 ^a,b^	1.86 ± 0.16 ^a,b^	2.35 ± 0.25 ^a,b^	3.41 ± 0.71 ^a,b^	0.54 ± 0.15 ^a,b^	2.57 ± 0.42 ^a,b^
UV + Elaea (50)	2.24 ± 0.20 ^a,b^	2.20 ± 0.22 ^a,b^	2.65 ± 0.26 ^a,c^	4.06 ± 0.23 ^a,c^	0.41 ± 0.05 ^a,b,e^	3.02 ± 0.21 ^a,b^

^a^ *p* < 0.01 versus control group; ^b^ *p* < 0.01, ^c^ *p* < 0.05 versus UV group; ^d^ *p* < 0.01, ^e^ *p* < 0.05 versus UV + AA group; *gsr*—*glutathione reductase*; *nox*—*NADPH oxidoreductase*; *mapk*—*mitogen-activated protein kinase*.

## Data Availability

Data is contained within the article and supplementary material.

## Supplementary Materials

The following supporting information can be downloaded at: https://www.mdpi.com/article/10.3390/antiox13020195/s1, Table S1: Chemical profiling of Elaea by using an UPLC-QTOF/MS.
