# Supplementary material for: Elaeagnus umbellata Fruit Extract Protects Skin from Ultraviolet-Mediated Photoaging in Hairless Mice
Source: Antioxidants (Basel). 2024 Feb 3;13(2):195. doi: 10.3390/antiox13020195 (PMC10885948; doi:10.3390/antiox13020195)
Supplement: Supplementary file 1 [file antioxidants-13-00195-s001.zip › antioxidants-2785429-supplementary.pdf]

antioxidants

MDPI

Supplementary Information

Elaeagnus umbellata Fruit Extract Protects Skin from Ultraviolet-Mediated Photoaging in Hairless Mice

Seok-Man Park [1,2], Cheol-Jong Jung [1,2], Dae-Geon Lee [1,2], Yeong-Eun Yu [2], Tae-Hun Ku [3], Mu-Seok Hong [4], Tae-Kyung Lim [4], Kwong-Il Paeng [4], Hyun-Ki Cho [4], Il-Je Cho [2,*] and Sae-Kwang Ku [1,*]

[1] Department of Histology and Anatomy, College of Korean Medicine, Daegu Haany University, Gyeongsan 38610, Republic of Korea; smpark@okchundang.co.kr (S.-M.P.); oc_cjjung@okchundang.co.kr (C.-J.J.); ghost71715@okchundang.co.kr (D.-G.L.)
[2] Central Research Center, Okchundang Inc., Daegu 41059, Republic of Korea; youye@okchundang.co.kr
[3] Okchundang Korean Medicine Clinic, Ulsan 44900, Republic of Korea; oc100002@okchundang.co.kr
[4] Rodam Korean Medical Clinic, Seoul 06038, Republic of Korea; hongms@skincora.com (M.-S.H.); hltkn@skincora.com (T.-K.L.); rodamca@skincora.com (K.-I.P.); wasavihg@skincora.com (H.-K.C.)
* Correspondence: oc100119@okchundang.co.kr (I.-J.C.); gucci200@dhu.ac.kr (S.-K.K.); Tel.: +82-53-950-0011 (I.-J.C.); +82-53-819-1549 (S.-K.K.)

1. Supplementary Materials and Methods

1.1. Extraction of Elaea

Elaea in 50% methanol (100 mg/mL) was sonicated at 30 °C for 20 min and then centrifuged at 12000 rpm for 10 min at 4 °C. The supernatant was filtered using a 0.22 µm polyvinylidene fluoride syringe filter.

1.2. UPLC-QTOF/MS analysis

Elaea was analyzed using a Nexera XS ultra performance liquid chromatography (UPLC) system (Shimadzu; Kyoto, Japan) coupled to an X500R quadrupole time-of-flight (QTOF)/mass spectrometry (MS)(SCIEX ExionLC AD system; Framingham, MA, USA) with an electrospray ionization source (ESI). A CAPCELL PAK UG120 C18 column (size, 250 × 4.6 mm; particle size, 5 µm)(Osaka soda; Osaka, Japan) was used for UPLC with a column oven temperature 35 °C. The flow rate was 0.5 mL/min, and the injection volume was 5 µL. The mobile phase was 0.1% formic acid in H_2O (solution A) and 0.1% formic acid in acetonitrile (solution B). The gradient elution was as follows: from 10 to 20% solution B for 0–8 min; from 20 to 50% solution B for 8–30 min; from 50 to 95% solution B for 30–50 min. The identification of compound using QTOF/MS was performed to both positive (ESI^+, 5500 V) and negative (ESI^-, -4500 V) ion modes. The conditions of MS were operated as follows: the ion source gas1 and gas2, 50 and 60 psi; the curtain gas, 30 psi; the ion source temperature, 550 °C. The mass spectra were recorded in the mass-to-charge (m/z) range of 100–1500 for QTOF/MS and 50–1500 for MS/MS with collision energy (CE 45 V, CE spread 15 V). Data acquisition and compound identification were performed using SCIEX OS software version 3.0.0.3339.

Table S1. Chemical profiling of Elaea by using an UPLC-QTOF/MS.

No.	Compound	Molecular formula	Adduct/Charge	Retention time (min)	Precursor mass (m/z)	Found at mass (m/z)	MS Fragment (m/z)
1	L-Alpha-aminobutyric acid	$C_4H_9NO_2$	[M+H]+	4.54	104.0707	104.0707	104, 58
2	Trigonelline	$C_7H_7NO_2$	[M+H]+	5.39	138.0554	138.0554	94, 92, 78
3	Pipecolinic acid	$C_6H_{11}NO_2$	[M+H]+	5.45	130.0868	130.0867	84, 67, 56

4	(-)-Quinic acid	$C_7H_{12}O_6$	[2M-H]-	5.65	383.1202	383.1196	191, 127, 85
			[2M+H]+	5.70	385.1362	385.1367	139, 111, 93
5	Shikimic acid	$C_7H_{10}O_5$	[M-H]-	5.78	173.0465	173.0465	93, 81, 71
6	maleic acid	$C_4H_4O_4$	[M-H]-	5.99	115.0040	115.0040	71
7	Gallic acid	$C_7H_6O_5$	[M+H]+	8.03	171.0294	171.0292	109, 107, 81
			[M-H]-	8.04	169.0141	169.0140	125, 124, 79
8	Coumarin	$C_9H_6O_2$	[M+H]+	8.16	147.0447	147.0446	91, 77, 65
9	1,2,3-Benzenetriol	$C_6H_6O_3$	[M-H]-	9.13	125.0252	125.0251	124, 95, 79
10	Ethylmalonic acid	$C_5H_8O_4$	[M-H]-	9.29	131.0358	131.0358	87
11	5-Hydroxymethylfurfural	$C_6H_6O_3$	[M+H]+	9.43	127.0384	127.0383	109, 81, 53
12	(-)-Gallocatechin	$C_{15}H_{14}O_7$	[M-H]-	9.45	305.0671	305.0669	167, 137, 125
			[M+H]+	9.47	307.0826	307.0825	163, 139
13	3-Indoleacrylic acid	$C_{11}H_9NO_2$	[M+H]+	9.57	188.0710	188.0709	170, 143, 118
14	2-Pyrocatechuic acid	$C_7H_6O_4$	[M+H]+	9.68	155.0344	155.0345	109, 81
15	Corilagin	$C_{27}H_{22}O_{18}$	[M-H]-	9.98	633.0761	633.0757	301, 275
16	Protocatechuic acid	$C_7H_6O_4$	[M-H]-	10.71	153.0193	153.0191	109, 108
17	3-O-Methylgallic acid	$C_8H_8O_5$	[M-H]-	11.32	183.0306	183.0305	168, 124, 123
			[M+H]+	11.42	185.0454	185.0455	126, 109, 81
18	4-Hydroxyquinoline	C_9H_7NO	[M+H]+	11.37	146.0606	146.0606	118, 104, 91
19	2-Furoic acid	$C_5H_4O_3$	[M-H]-	12.21	111.0094	111.0094	67, 65
20	1-Naphthalenamine	$C_{10}H_9N$	[M+H]+	12.39	144.0812	144.0810	143, 127, 115
21	Epicatechin	$C_{15}H_{14}O_6$	[M-H]-	12.60	289.0730	289.0728	149, 123, 109
22	Catechin	$C_{15}H_{14}O_6$	[M+H]+	12.61	291.0877	291.0876	147, 139, 123
23	3,4-Dihydroxybenzaldehyde	$C_7H_6O_3$	[M-H]-	13.02	137.0245	137.0245	137, 136, 108
24	Salicyclic acid	$C_7H_6O_3$	[M-H]-	13.80	137.0251	137.0251	93, 65
25	Esculetin	$C_9H_6O_4$	[M-H]-	14.53	177.0205	177.0203	105, 89, 77
26	Ethyl gallate	$C_9H_{10}O_5$	[M-H]-	15.23	197.0462	197.0464	123, 95, 78
27	Syringic acid	$C_9H_{10}O_5$	[M+H]+	15.24	199.0611	199.0610	140, 125, 97
28	1,2-Benzenedicarboxylic acid	$C_8H_6O_4$	[M-H]-	15.35	165.0204	165.0203	121, 77
29	Dihydromyricetin	$C_{15}H_{12}O_8$	[M-H]-	15.81	319.0472	319.0471	193, 125, 57
30	Robinin	$C_{33}H_{40}O_{19}$	[M-H]-	16.34	739.2109	739.2104	739, 593, 430
			[M+H]+	16.35	741.2253	741.2250	433, 287
31	3-Hydroxybenzaldehyde	$C_7H_6O_2$	[M-H]-	16.66	121.0296	121.0295	121, 93, 92
32	4-Hydroxybenzaldehyde	$C_7H_6O_2$	[M+H]+	16.66	123.0446	123.0446	95, 77, 51
33	Rutin	$C_{27}H_{30}O_{16}$	[M-H]-	17.72	609.1470	609.1466	609, 301, 300
			[M+H]+	17.74	611.1641	611.1636	303, 85
34	Phloroglucinocarboxaldehyde	$C_7H_6O_4$	[M-H]-	18.10	153.0201	153.0200	151, 83, 65
35	Polydatin	$C_{20}H_{22}O_8$	[M+HCOOH-H]-	18.39	435.1312	435.1314	227, 185
36	*p*-Coumaric acid	$C_9H_8O_3$	[M-H]-	18.39	163.0409	163.0408	119, 117, 93
37	trans-3-Coumaric acid	$C_9H_8O_3$	[M+H]+	18.39	165.0556	165.0556	147, 119, 91
38	Ellagic acid	$C_{14}H_6O_8$	[M+H]+	18.77	303.0134	303.0132	303, 257, 201

			[M-H]-	18.77	300.9987	300.9986	300, 283, 145
39	Aempferol-3-O-rutinoside	$C_{27}H_{30}O_{15}$	[M-H]-	18.96	593.1533	593.1534	593, 285, 284
			[M+H]+	18.97	595.1675	595.1679	287, 85
40	Astragalin	$C_{21}H_{20}O_{11}$	[M-H]-	20.56	447.0940	447.0936	285, 284, 255
			[M+H]+	20.58	449.1089	449.1085	287, 153, 85
41	Myricetin	$C_{15}H_{10}O_8$	[M-H]-	22.66	317.0314	317.0313	178, 151, 137
			[M+H]+	22.67	319.0462	319.0462	319, 217, 153
42	Quercetin 7-rhamnoside ; Vincetoxicoside B	$C_{21}H_{20}O_{11}$	[M-H]-	23.05	447.0932	447.0933	301, 300, 151
43	Rhodionin	$C_{21}H_{20}O_{11}$	[M+H]+	23.06	449.1077	449.1073	303, 229
44	4-Hydroxybenzoic acid	$C_7H_6O_3$	[M-H]-	24.69	137.0249	137.0248	93, 65
45	Tiliroside	$C_{30}H_{26}O_{13}$	[M-H]-	26.07	593.1313	593.1309	593, 285, 284
			[M+H]+	26.08	595.1455	595.1450	309, 287, 147
46	Afzelin	$C_{21}H_{20}O_{10}$	[M+H]+	26.10	433.1133	433.1129	287, 153
47	Calycosin-7-o-glucoside	$C_{22}H_{22}O_{10}$	[M+HCOOH-H]-	26.76	491.1209	491.1206	283, 268
48	Glycitin	$C_{22}H_{22}O_{10}$	[M+H]+	26.76	447.1298	447.1295	285, 242
49	Morin	$C_{15}H_{10}O_7$	[M+H]+	26.78	303.0510	303.0509	303, 137, 68
50	Quercetin	$C_{15}H_{10}O_7$	[M-H]-	26.79	301.0362	301.0360	178, 151, 121
51	Kaempferol	$C_{15}H_{10}O_6$	[M-H]-	30.67	285.0415	285.0413	145, 108, 93
			[M+H]+	30.69	287.0562	287.0561	287, 153, 121
52	Tamarixetin	$C_{16}H_{12}O_7$	[M-H]-	31.26	315.0527	315.0524	315, 300, 151
53	Asiatic acid	$C_{30}H_{48}O_5$	[M-H]-	39.37	487.3440	487.3443	487
			[M+NH4]+	39.38	506.3856	506.3856	435, 407, 201
54	Decursin	$C_{19}H_{20}O_5$	[M+H]+	43.60	329.1393	329.1391	229, 214, 213
